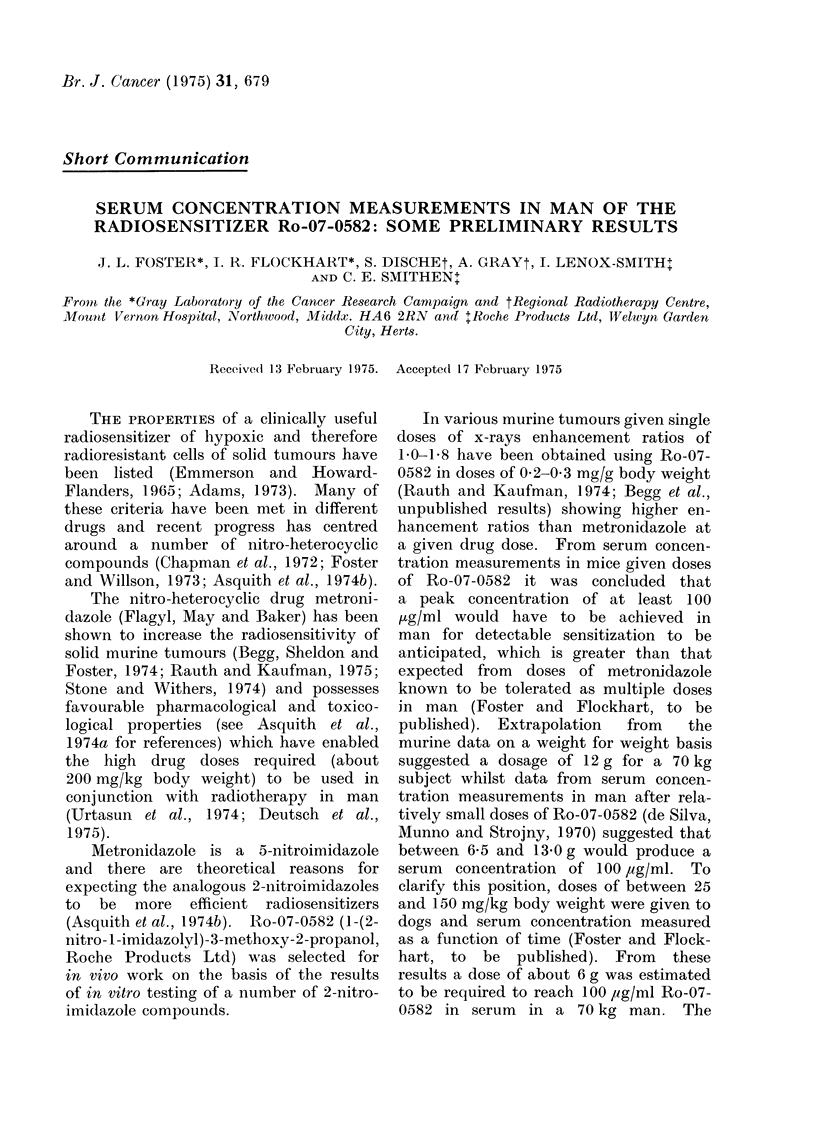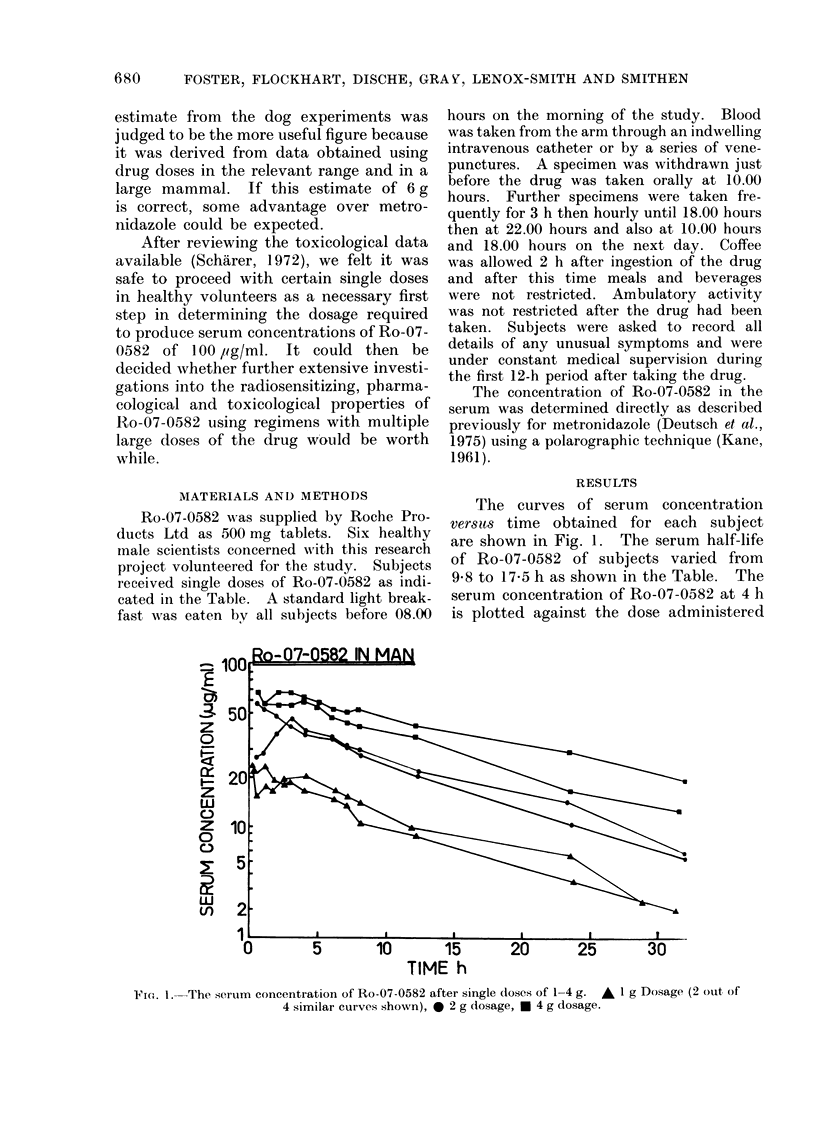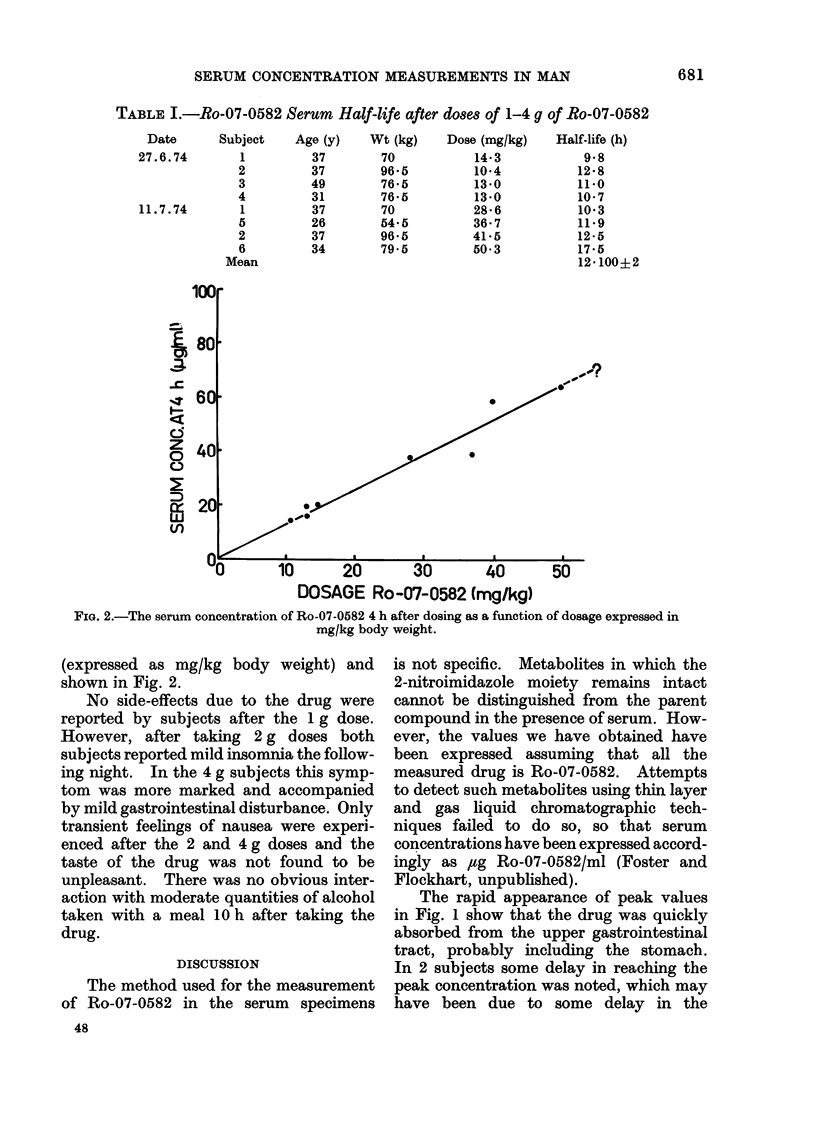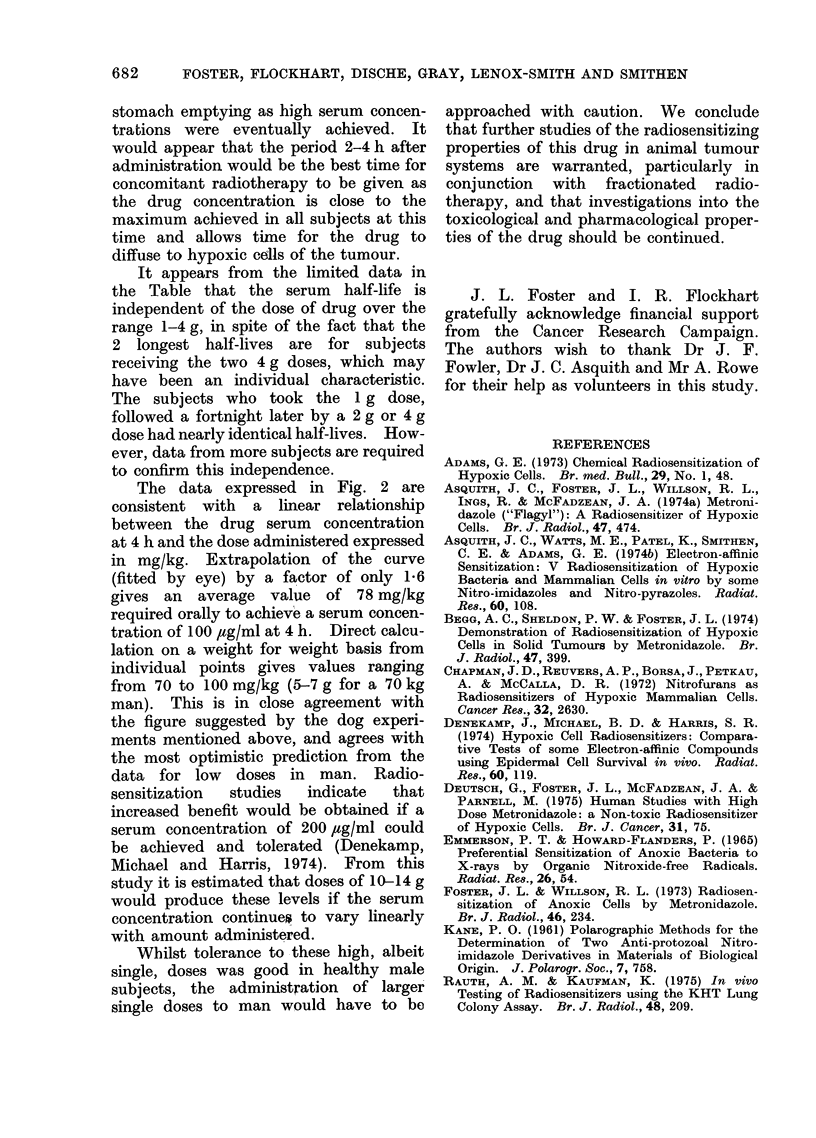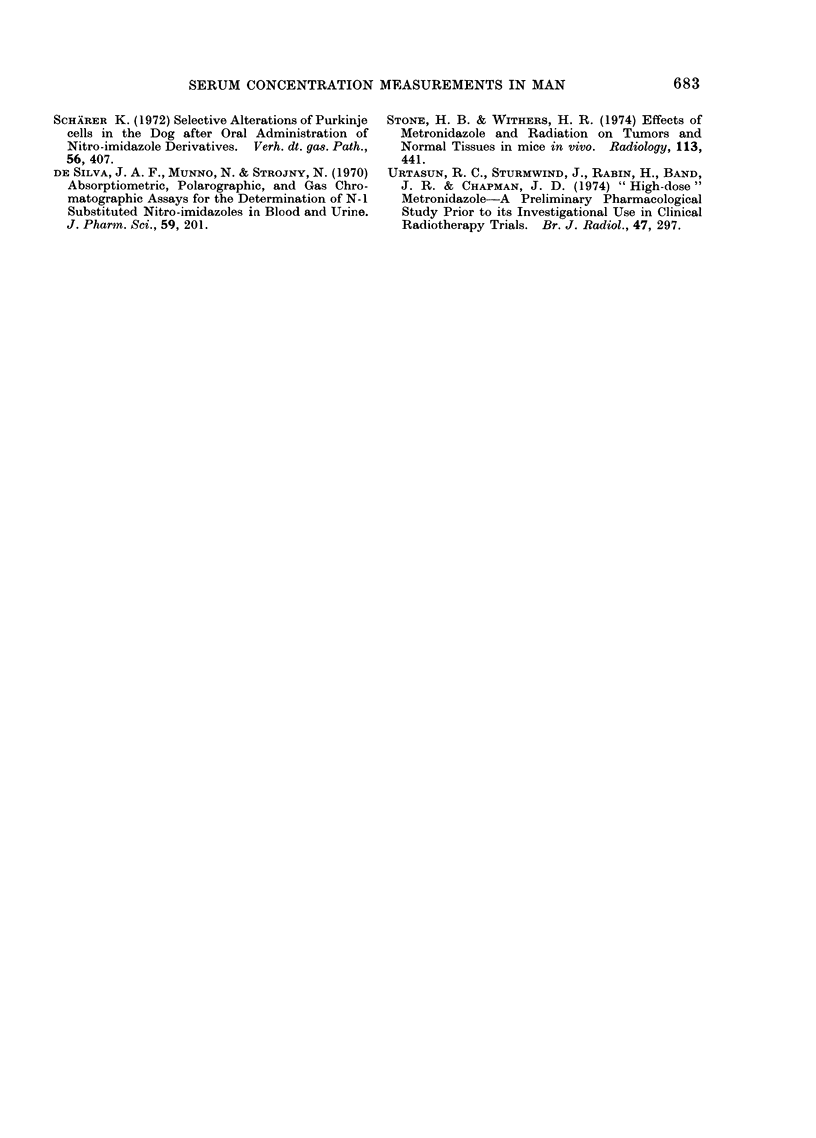# Serum concentration measurements in man of the radiosensitizer Ro-07-0582: some preliminary results.

**DOI:** 10.1038/bjc.1975.115

**Published:** 1975-06

**Authors:** J. L. Foster, I. R. Flockhart, S. Dische, A. Gray, I. Lenox-Smith, C. E. Smithen


*Br. J. Cancer* (1975) **31**, 679

***Short Communication***

### SERUM CONCENTRATION MEASUREMENTS IN MAN OF THE RADIOSENSITIZER Ro-07-0582: SOME PRELIMINARY RESULTS

J. L. FOSTER*, I. R. FLOCKHART*, S. DISCHE†, A. GRAY†, I. LENOX-SMITH‡ AND C. E. SMITHEN‡

*From the *Gray Laboratory of the Cancer Research Campaign and †Regional Radiotherapy Centre, Mount Vernon Hospital, Northwood, Middx. HA6 2RN and ‡Roche Products Ltd, Welwyn Garden City, Herts.*

Received 13 February 1975. Accepted 17 February 1975

THE PROPERTIES of a clinically useful radiosensitizer of hypoxic and therefore radioresistant cells of solid tumours have been listed (Emmerson and Howard-Flanders, 1965; Adams, 1973). Many of these criteria have been met in different drugs and recent progress has centred around a number of nitro-heterocyclic compounds (Chapman *et al.*, 1972; Foster and Willson, 1973; Asquith *et al.*, 1974*b*).

The nitro-heterocyclic drug metronidazole (Flagyl, May and Baker) has been shown to increase the radiosensitivity of solid murine tumours (Begg, Sheldon and Foster, 1974; Rauth and Kaufman, 1975; Stone and Withers, 1974) and possesses favourable pharmacological and toxicological properties (see Asquith *et al.*, 1974*a* for references) which have enabled the high drug doses required (about 200 mg/kg body weight) to be used in conjunction with radiotherapy in man (Urtasun *et al.*, 1974; Deutsch *et al.*, 1975).

Metronidazole is a 5-nitroimidazole and there are theoretical reasons for expecting the analogous 2-nitroimidazoles to be more efficient radiosensitizers (Asquith *et al.*, 1974*b*). Ro-07-0582 (1-(2-nitro-1-imidazolyl)-3-methoxy-2-propanol, Roche Products Ltd) was selected for *in vivo* work on the basis of the results of *in vitro* testing of a number of 2-nitroimidazole compounds.

In various murine tumours given single doses of x-rays enhancement ratios of 1·0–1·8 have been obtained using Ro-07-0582 in doses of 0·2–0·3 mg/g body weight (Rauth and Kaufman, 1974; Begg *et al.*, unpublished results) showing higher enhancement ratios than metronidazole at a given drug dose. From serum concentration measurements in mice given doses of Ro-07-0582 it was concluded that a peak concentration of at least 100 μg/ml would have to be achieved in man for detectable sensitization to be anticipated, which is greater than that expected from doses of metronidazole known to be tolerated as multiple doses in man (Foster and Flockhart, to be published). Extrapolation from the murine data on a weight for weight basis suggested a dosage of 12 g for a 70 kg subject whilst data from serum concentration measurements in man after relatively small doses of Ro-07-0582 (de Silva, Munno and Strojny, 1970) suggested that between 6·5 and 13·0 g would produce a serum concentration of 100 μg/ml. To clarify this position, doses of between 25 and 150 mg/kg body weight were given to dogs and serum concentration measured as a function of time (Foster and Flockhart, to be published). From these results a dose of about 6 g was estimated to be required to reach 100 μg/ml Ro-07-0582 in serum in a 70 kg man. The estimate from the dog experiments was judged to be the more useful figure because it was derived from data obtained using drug doses in the relevant range and in a large mammal. If this estimate of 6 g is correct, some advantage over metronidazole could be expected.

After reviewing the toxicological data available (Schärer, 1972), we felt it was safe to proceed with certain single doses in healthy volunteers as a necessary first step in determining the dosage required to produce serum concentrations of Ro-07-0582 of 100 μg/ml. It could then be decided whether further extensive investigations into the radiosensitizing, pharmacological and toxicological properties of Ro-07-0582 using regimens with multiple large doses of the drug would be worth while.

### MATERIALS AND METHODS

Ro-07-0582 was supplied by Roche Products Ltd as 500 mg tablets. Six healthy male scientists concerned with this research project volunteered for the study. Subjects received single doses of Ro-07-0582 as indicated in the Table. A standard light breakfast was eaten by all subjects before 08.00 hours on the morning of the study. Blood was taken from the arm through an indwelling intravenous catheter or by a series of venepunctures. A specimen was withdrawn just before the drug was taken orally at 10.00 hours. Further specimens were taken frequently for 3 h then hourly until 18.00 hours then at 22.00 hours and also at 10.00 hours and 18.00 hours on the next day. Coffee was allowed 2 h after ingestion of the drug and after this time meals and beverages were not restricted. Ambulatory activity was not restricted after the drug had been taken. Subjects were asked to record all details of any unusual symptoms and were under constant medical supervision during the first 12-h period after taking the drug.

The concentration of Ro-07-0582 in the serum was determined directly as described previously for metronidazole (Deutsch *et al.*, 1975) using a polarographic technique (Kane, 1961).

### RESULTS

The curves of serum concentration *versus* time obtained for each subject are shown in Fig. 1. The serum half-life of Ro-07-0582 of subjects varied from 9·8 to 17·5 h as shown in the Table. The serum concentration of Ro-07-0582 at 4 h is plotted against the dose administered

FIG. 1.—The serum concentration of Ro-07-0582 after single doses of 1–4 g. ▲ 1 g Dosage (2 out of 4 similar curves shown), ● 2 g dosage, ■ 4 g dosage.

TABLE I.—*Ro*-07-0582 *Serum Half-life after doses of* 1–4 *g of Ro*-07-0582

| Date | Subject | Age (y) | Wt (kg) | Dose (mg/kg) | Half-life (h) |
|---|---|---|---|---|---|
| 27.6.74 | 1 | 37 | 70 | 14·3 | 9·8 |
| | 2 | 37 | 96·5 | 10·4 | 12·8 |
| | 3 | 49 | 76·5 | 13·0 | 11·0 |
| | 4 | 31 | 76·5 | 13·0 | 10·7 |
| 11.7.74 | 1 | 37 | 70 | 28·6 | 10·3 |
| | 5 | 26 | 54·5 | 36·7 | 11·9 |
| | 2 | 37 | 96·5 | 41·5 | 12·5 |
| | 6 | 34 | 79·5 | 50·3 | 17·5 |
| | Mean | | | | 12·100±2 |

FIG. 2.—The serum concentration of Ro-07-0582 4 h after dosing as a function of dosage expressed in mg/kg body weight.

(expressed as mg/kg body weight) and shown in Fig. 2.

No side-effects due to the drug were reported by subjects after the 1 g dose. However, after taking 2 g doses both subjects reported mild insomnia the following night. In the 4 g subjects this symptom was more marked and accompanied by mild gastrointestinal disturbance. Only transient feelings of nausea were experienced after the 2 and 4 g doses and the taste of the drug was not found to be unpleasant. There was no obvious interaction with moderate quantities of alcohol taken with a meal 10 h after taking the drug.

### DISCUSSION

The method used for the measurement of Ro-07-0582 in the serum specimens is not specific. Metabolites in which the 2-nitroimidazole moiety remains intact cannot be distinguished from the parent compound in the presence of serum. However, the values we have obtained have been expressed assuming that all the measured drug is Ro-07-0582. Attempts to detect such metabolites using thin layer and gas liquid chromatographic techniques failed to do so, so that serum concentrations have been expressed accordingly as µg Ro-07-0582/ml (Foster and Flockhart, unpublished).

The rapid appearance of peak values in Fig. 1 show that the drug was quickly absorbed from the upper gastrointestinal tract, probably including the stomach. In 2 subjects some delay in reaching the peak concentration was noted, which may have been due to some delay in the stomach emptying as high serum concentrations were eventually achieved. It would appear that the period 2–4 h after administration would be the best time for concomitant radiotherapy to be given as the drug concentration is close to the maximum achieved in all subjects at this time and allows time for the drug to diffuse to hypoxic cells of the tumour.

It appears from the limited data in the Table that the serum half-life is independent of the dose of drug over the range 1–4 g, in spite of the fact that the 2 longest half-lives are for subjects receiving the two 4 g doses, which may have been an individual characteristic. The subjects who took the 1 g dose, followed a fortnight later by a 2 g or 4 g dose had nearly identical half-lives. However, data from more subjects are required to confirm this independence.

The data expressed in Fig. 2 are consistent with a linear relationship between the drug serum concentration at 4 h and the dose administered expressed in mg/kg. Extrapolation of the curve (fitted by eye) by a factor of only 1·6 gives an average value of 78 mg/kg required orally to achieve a serum concentration of 100 $\mu$g/ml at 4 h. Direct calculation on a weight for weight basis from individual points gives values ranging from 70 to 100 mg/kg (5–7 g for a 70 kg man). This is in close agreement with the figure suggested by the dog experiments mentioned above, and agrees with the most optimistic prediction from the data for low doses in man. Radiosensitization studies indicate that increased benefit would be obtained if a serum concentration of 200 $\mu$g/ml could be achieved and tolerated (Denekamp, Michael and Harris, 1974). From this study it is estimated that doses of 10–14 g would produce these levels if the serum concentration continues to vary linearly with amount administered.

Whilst tolerance to these high, albeit single, doses was good in healthy male subjects, the administration of larger single doses to man would have to be approached with caution. We conclude that further studies of the radiosensitizing properties of this drug in animal tumour systems are warranted, particularly in conjunction with fractionated radiotherapy, and that investigations into the toxicological and pharmacological properties of the drug should be continued.

J. L. Foster and I. R. Flockhart gratefully acknowledge financial support from the Cancer Research Campaign. The authors wish to thank Dr J. F. Fowler, Dr J. C. Asquith and Mr A. Rowe for their help as volunteers in this study.

### REFERENCES

Adams, G. E. (1973) Chemical Radiosensitization of Hypoxic Cells. *Br. med. Bull.*, **29**, No. 1, 48.

Asquith, J. C., Foster, J. L., Willson, R. L., Ings, R. & McFadzean, J. A. (1974a) Metronidazole ("Flagyl"): A Radiosensitizer of Hypoxic Cells. *Br. J. Radiol.*, **47**, 474.

Asquith, J. C., Watts, M. E., Patel, K., Smithen, C. E. & Adams, G. E. (1974*b*) Electron-affinic Sensitization: V Radiosensitization of Hypoxic Bacteria and Mammalian Cells *in vitro* by some Nitro-imidazoles and Nitro-pyrazoles. *Radiat. Res.*, **60**, 108.

Begg, A. C., Sheldon, P. W. & Foster, J. L. (1974) Demonstration of Radiosensitization of Hypoxic Cells in Solid Tumours by Metronidazole. *Br. J. Radiol.*, **47**, 399.

Chapman, J. D., Reuvers, A. P., Borsa, J., Petkau, A. & McCalla, D. R. (1972) Nitrofurans as Radiosensitizers of Hypoxic Mammalian Cells. *Cancer Res.*, **32**, 2630.

Denekamp, J., Michael, B. D. & Harris, S. R. (1974) Hypoxic Cell Radiosensitizers: Comparative Tests of some Electron-affinic Compounds using Epidermal Cell Survival *in vivo*. *Radiat. Res.*, **60**, 119.

Deutsch, G., Foster, J. L., McFadzean, J. A. & Parnell, M. (1975) Human Studies with High Dose Metronidazole: a Non-toxic Radiosensitizer of Hypoxic Cells. *Br. J. Cancer*, **31**, 75.

Emmerson, P. T. & Howard-Flanders, P. (1965) Preferential Sensitization of Anoxic Bacteria to X-rays by Organic Nitroxide-free Radicals. *Radiat. Res.*, **26**, 54.

Foster, J. L. & Willson, R. L. (1973) Radiosensitization of Anoxic Cells by Metronidazole. *Br. J. Radiol.*, **46**, 234.

Kane, P. O. (1961) Polarographic Methods for the Determination of Two Anti-protozoal Nitroimidazole Derivatives in Materials of Biological Origin. *J. Polarogr. Soc.*, **7**, 758.

Rauth, A. M. & Kaufman, K. (1975) *In vivo* Testing of Radiosensitizers using the KHT Lung Colony Assay. *Br. J. Radiol.*, **48**, 209.

SCHÄRER K. (1972) Selective Alterations of Purkinje cells in the Dog after Oral Administration of Nitro-imidazole Derivatives. *Verh. dt. gas. Path.*, **56**, 407.

DE SILVA, J. A. F., MUNNO, N. & STROJNY, N. (1970) Absorptiometric, Polarographic, and Gas Chromatographic Assays for the Determination of N-1 Substituted Nitro-imidazoles in Blood and Urine. *J. Pharm. Sci.*, **59**, 201.

STONE, H. B. & WITHERS, H. R. (1974) Effects of Metronidazole and Radiation on Tumors and Normal Tissues in mice *in vivo*. *Radiology*, **113**, 441.

URTASUN, R. C., STURMWIND, J., RABIN, H., BAND, J. R. & CHAPMAN, J. D. (1974) "High-dose" Metronidazole—A Preliminary Pharmacological Study Prior to its Investigational Use in Clinical Radiotherapy Trials. *Br. J. Radiol.*, **47**, 297.